# Oral microbiome characteristics in patients with pediatric solid tumor

**DOI:** 10.3389/fmicb.2023.1286522

**Published:** 2024-01-05

**Authors:** Xichun Cui, Xiaoran Du, Xu Cui, Rongrong Fan, Juntao Pan, Zhifang Wang

**Affiliations:** ^1^Pediatric Surgery Department, The First Affiliated Hospital of Zhengzhou University, Zhengzhou, China; ^2^Endocrinology Department, The First Affiliated Hospital of Zhengzhou University, Zhengzhou University, Zhengzhou, Henan, China

**Keywords:** 16S rRNA sequencing, oral microbiome, pediatric solid tumor, healthy controls, microbial markers

## Abstract

**Background:**

Pediatric solid tumor, the abnormal proliferation of solid tissues in children resulting in the formation of tumors, represent a prevailing malignant ailment among the younger population. Extensive literature highlights the inseparable association linking oral microbiome and adult tumors, but due to differences in age of onset, characteristics of onset, etc., there are many differences between Pediatric solid tumors and adult tumors, and therefore, studying the relationship between Pediatric solid tumor and the oral microbiota is also essential.

**Methods:**

To unravel the distinct characteristics of the oral microbiota within Pediatric solid tumor patients, 43 saliva samples, encompassing 23 Pediatric solid tumor patients and 20 healthy controls, were diligently procured. A meticulous screening process ensued, and conducted microbial MiSeq sequencing after screening.

**Results:**

We documented the oral microbiome attributes among pediatric diagnosed with solid tumors (PST), and meanwhile, we observed a significant trend of decreased oral microbiota diversity in the pediatric solid tumor group. There were notable disparities in microbial communities observed between the two groups, 18 genera including Veillonellaceae, Firmicutes unclassified, Coriobacteriia, Atopobiaceae, Negativicutes, were significantly enriched in PST patients, while 29 genera, including Gammaproteobacteria, Proteobacteria, Burkholderiales, Neisseriaceae, were dominant in the HCs group. It was found that PST group had 16 gene functions, including Amino acid metabolism, Cysteine and methionine metabolism, Photosynthesis antenna proteins, Arginine and proline metabolism, and Aminoacyl tRNA biosynthesi, were significantly dominant, while 29 gene functions that prevailed in HCs.

**Conclusion:**

This study characterized the oral microbiota of Pediatric solid tumor patients for the first time, and importantly, targeted biomarkers of oral microbiota may serve as powerful and non-invasive diagnostic tools for pediatric solid tumor patients.

## Introduction

Childhood malignancies, ranking as the second most common cause of death in developed countries following accidents, Blattner-Johnson et al. (2022) have witnessed a gradual rise in occurrence over the last 30 years, with an annual rise of approximately 1% across all types of cancer combined (Steliarova-Foucher et al., 2018). This amounts to an estimated 400,000 cases per year worldwide (Ward et al., 2019). Childhood cancers, arising in the context of actively growing tissues, can be considered as diseases resulting from dysregulated development (Filbin and Monje, 2019). Pediatric solid tumors are characterized by the abnormal growth of solid tissues in children, leading to the formation of tumors. These tumors are prevalent among children and are considered one of the most common malignant diseases in this population. There are numerous types of solid tumors, including neuroblastoma, osteosarcoma, Ewing sarcoma, rhabdomyosarcoma, non-rhabdomyosarcoma, soft tissue sarcoma, liver tumors, kidney tumors, and extracranial germ cell tumors, among others (van Kalsbeek et al., 2023). Among them, neuroblastoma (NB) is the most common extracranial malignant solid tumor in children, accounting for 8–10% of all pediatric malignancies and 15% of all childhood cancer-related deaths (Maris, 2010). Osteosarcoma is the most common primary malignant bone tumor in children and adolescents (Eaton et al., 2021). Rhabdomyosarcoma (RMS) is the most common soft tissue sarcoma in children (Schloemer et al., 2023). Wilms tumor is the most common kidney tumor in childhood, accounting for 90% of cases (Bertacca et al., 2023). They exhibit a high metastatic potential, poor prognosis, and significant resistance to current therapeutic approaches (Aboushanab et al., 2022). Cancer remains one of the leading causes of death among children and adolescents (Steliarova-Foucher et al., 2017). Solid tumors in children are also attracting increasing attention. Cancer remains a notable public health concern, given its significant impact on the physical and emotional well-being of afflicted children and their families. Approximately 15,000 children and adolescents between the ages of 0 and 19 are diagnosed with cancer annually in the United States (Bhatia et al., 2023). For effective early detection and treatment, it is imperative that primary care physicians and parents are well-informed about the initial signs of childhood malignancies (Dang-Tan and Franco, 2007) when refer to diagnosis, it has been more challenging to develop tests for children (Nierengarten, 2023). Therefore, there is a need for a concise and efficient diagnostic tool for pediatric solid tumors, which is the purpose of our research.

The oral cavity serves as the initial entry point for the colonization of oral and gut microbiota (Derrien et al., 2010). Oral microbiota refers to the microbial community present in the oral cavity, encompassing bacteria, viruses, fungi and other microorganisms. There are over 700 species of bacteria that can be found in the human oral cavity (Paster et al., 2000). These microorganisms play a significant role in oral health, maintaining a dynamic balance with the host’s immune system and helping prevent the invasion of pathogenic microorganisms. The oral microbiota is the second most diverse microbial community after the gut microbiota (Tuganbaev et al., 2022). With the advancement in oral microbiota research, it has been discovered that the dysbiosis of oral bacterial communities is associated with the occurrence and development of various oral diseases, such as periodontitis, Topcuoglu and Kulekci (2015) oral leukoplakia (OLK), Amer et al. (2017) systemic lupus erythematosus (Corrêa et al., 2017). Oral inflammation can also potentially lead to systemic diseases through bacteremia (Han and Wang, 2013). Strains of Klebsiella bacteria from the salivary microbiota, when colonized in the gut, can effectively induce chronic intestinal inflammation (Atarashi et al., 2017). Recently, as oral microbiome research has progressed, an increasing recognition has emerged regarding the interconnectedness of cancer with the oral microbiome. For example, The makeup of the oral microbiome suggests a prospective susceptibility to developing esophageal cancers (Peters et al., 2017). There is a correlation between the oral microbiome and an elevated likelihood of developing new cases of head and neck squamous cell cancer (Hayes et al., 2018). In the past few decades, there has been a growing interest in studying the human microbiome and its role in health and disease. The focus of these studies has mainly been to characterize the variations in microbial community composition under different conditions (Solbiati and Frias-Lopez, 2018).

However, there have been limited studies on the oral microbiome of pediatric solid tumor patients so far. Therefore, it is crucial to investigate the oral microbiome in the field of pediatric solid tumors. This research delves into the oral microbiome traits among pediatric patients with solid tumors in central China. It underscores the promising prospect of leveraging the oral microbiome can be used for acting as a diagnostic instrument for pediatric solid tumors.

## Materials and methods

### Articipant InfoPrmation

The investigation was meticulously designed and carried out following the PRoBE (Retrospective Blinded Evaluation and Prospective Specimen Collection) guidelines, in alignment with the principles outlined in the Regulations of Good Clinical Practice and the Helsinki Declaration. Exclusively sourced from the First Affiliated Hospital of Zhengzhou University, the study specifically acquired saliva samples. All individuals enrolled in the research were recently diagnosed outpatients with pediatric solid tumors. The patients selected for this study have all been diagnosed as pediatric solid tumor patients through tissue biopsy and pathological examination. The participants consisted exclusively of patients newly diagnosed with pediatric solid tumors, who had not undergone any prior treatments. Exclusion criteria encompassed the following conditions: (Blattner-Johnson et al., 2022) Concurrent presence of other diseases, Steliarova-Foucher et al. (2018). Use of antibiotics within the past eight weeks, and (Ward et al., 2019) medical history includes tumor resection or previous treatments such as radiotherapy and chemotherapy. In the context of this research, a grand total of 50 saliva samples were gathered. These encompassed 25 samples from pediatric solid tumor (PST) patients and an additional 25 samples from individuals designated as healthy controls (HCs). Following rigorous screening, 23 pediatric solid tumor (PST) patients and 20 healthy controls (HCs), with age, sex, and BMI matching, were included in the study. Ultimately, 43 saliva samples from pediatric solid tumor patients and healthy controls underwent 16S rRNA MiSeq sequencing.

Clinicopathological and demographic information of participants was gathered from questionnaires and hospital electronic medical records (Table 1).

**Table 1:** Demographic characteristics of patients with Pediatric solid tumor and the age- and gender-matched healthy controls.

Characteristics	PST	HC
*N*	23	20
*Age (years)*	5.1 [1.0, 7.0]	5.1 [2.0, 11.0]
*Gender*		
Male	14 (60.9%)	20 (100%)
Female	9 (39.1%)	0
*BMI (kg/m2)*	18.7 [14.6, 21.8]	16.7 [14.8, 20.9]
*Disease type*		
Neurogenic tumor	7 (30.4%)	/
Teratoma	5 (21.8%)	/
Sarcoma	4 (17.4%)	/
Other tumors	7 (30.4%)	/
*Any other comorbidities*	0	0
*History of surgery*	0	0
*radiotherapy or chemotherapy*	0	0
*Use of antibiotics*	0	0

Data are presented as a number with the percentage in parentheses, or as the median with the interquartile range (IQR) in square brackets. Pediatric solid tumor (PST), healthy controls (HCs).

### Saliva sample collection

Collect and preprocess saliva samples following the procedures described in previous reports (Bajaj et al., 2015). Saliva samples should be collected promptly upon enrollment to minimize potential influences from medical interventions that could alter the saliva microbiome. Participants were guided to perform teeth brushing twice a day for the purpose of maintaining optimal oral hygiene, both in the morning and before bedtime. Prior to supplying saliva samples, every participant was recommended to refrain from consuming any food or beverages for a duration of 2 h. Saliva samples were collected into specific collection tubes, with each individual requiring approximately 5 mL. The specimens were promptly preserved at a temperature of −80°C. All study participants refrained from consuming probiotics, antibiotics, cigarettes, or drugs for a period of eight weeks prior to enrollment. Genomic DNA extraction was performed utilizing the DNA extraction kit, following the methodology described in previous reports (Ren et al., 2019).

### DNA extraction, PCR amplification, and MiSeq sequencing

Uniform methods for DNA extraction and PCR amplification were applied to all samples, with the processes being carried out consistently by the laboratory personnel. The sample was suspended in 790 μL of sterile lysis buffer (4 M guanidine thiocyanate; 10% N-lauroyl sarcosine; 5% N-lauroyl sarcosine-0.1 M phosphate buffer [pH 8.0]) in 2 mL screw-cap tube containing 1 g glass beads (0.1 mm BioSpec Products, Inc., United States). The combination was subjected to vigorous vortexing and subsequently placed in an incubator at a temperature of 70°C for a duration of 1 h. Following this, the mixture underwent bead beating for 10 min at the highest speed. Bacterial DNA extraction was performed as per the guidelines provided by the manufacturer, utilizing The E.Z.N.A.® DNA Kit (Omega Bio-tek, Inc., GA). The extracted DNA was then stored at −20°C for subsequent analysis. Utilizing the DNA extracted from each sample, we employed it as a blueprint to magnify the V3 ~ V4 region within the 16S rRNA genes. The V3 ~ V4 region of each sample’s 16S rRNA gene, spanning positions 341 to 805 in the *Escherichia coli* genome, was amplified using PCR with the primers F1 and R2 (5’-GACTACHVGGGTATCTAATCC-3′ and 5’-CCTACGGGNGGCWGCAG-3′). PCR reactions were conducted in an EasyCycler 96 PCR system (Analytik Jena Corp., AG) using the subsequent protocol: an initial denaturation of 3 min at 95°C, ensued by 21 cycles comprising a 0.5-min denaturation at 94°C, a 0.5-min annealing at 58°C, and a 0.5-min elongation at 72°C. The process concluded with a final extension at 72°C for 5 min. The amplified outputs derived from various samples were assigned indexes and blended in equidistant proportions to facilitate sequencing. Shanghai Mobio Biomedical Technology Co. Ltd., following the guidelines of the manufacturer, conducted the sequencing process on the Miseq platform (Illumina Inc., United States).

### Sequence data processing

After the selected readings were sorted, they were allocated to distinct sample categories through the utilization of specific barcodes. Subsequently, the barcodes and primers were eliminated. The paired-end sequences originating from each library were combined using FLASH v. 1.2.10, employing the default parameters to facilitate overlap (Magoč and Salzberg, 2011). The combined reads resulting from the FLASH process underwent quality assessment, wherein UCHIME v. 4.2.40 was employed to identify and eliminate any chimera sequences (Edgar et al., 2011). The nucleotide sequences from all samples were uploaded to the National Center for Biotechnology Information (NCBI) database in the United States (PRJNA991748).

### OTU clustering and taxonomic annotation

A randomized selection of an equal number of reads was performed across all samples, Subsequently, the operational taxonomic units (OTUs) were categorized using the UPARSE pipeline (Edgar, 2013). We calculated the total number of operational taxonomic units (OTUs) at various taxonomic levels, including phylum, class, order, family, and genus. The statistical table displays the OTU serial numbers for all samples (Kakiyama et al., 2013).

### Bacterial diversity and taxonomic analysis

Bacterial community diversity was gaged through the computation of Simpson and Shannon indices, facilitated by the “vegan” R package. Meanwhile, the abundance of the bacterial community was estimated utilizing the Ace and Chao estimators. Venn diagrams were employed to visually represent the likeness and convergence of operational taxonomic units, revealing shared and unique units across various samples. Additionally, Heatmap Builder was utilized to generate heatmaps, providing a visual representation of dominant species. Through species composition analysis, we created bar plots representing the microbial community. The ‘vegan’ R package was employed to conduct NMDS (Non-Metric Multidimensional Scaling) and Principal Coordinates Analysis (PCoA), with the aim of elucidating the microbial dissimilarity among samples. For identification purposes, the “phyloseq” package was applied to compute unweighted and weighted UniFrac distances. Phylogenetic trees were employed to illustrate the evolutionary connections among bacteria. Taxonomic analyses of bacteria were performed across multiple hierarchical levels, encompassing phylum, class, order, family, and genus. Subsequently, to assess microbiome distinctions between the two groups, we conducted Wilcoxon rank-sum tests. Additionally, the linear discriminant analysis effect size (LEfSe) technique was utilized for conducting linear discriminant analysis (LDA) and pinpointing pivotal microbiomes that displayed notable dissimilarities. The analysis was conducted using the LEfSe online tool available at http://huttenhower.sph.harvard.edu/lefse/e/. A threshold was determined at an LDA (linear discriminant analysis) score of log10 = 2 (Lu et al., 2017). Additionally, key biomarkers (community members) were identified using both the nonparametric Kruskal-Wallis rank-sum test and the Wilcoxon rank-sum test.

### Statistical analysis

The data was analyzed using SPSS v. 20.0 (IBM Corp., Armonk, NY, United States). We calculated the statistical significance of disparities between groups. Fisher’s exact test was employed for the comparison of categorical variables, whereas the Wilcoxon rank-sum test was used for continuous variables. For correlation analysis, we utilized Spearman’s rank test.

### Gene function prediction

We utilized PICRUSt to forecast the gene functionalities of both 16S rRNA gene sequences and the oral microbiome. This was achieved through referencing the KEGG (Kyoto Encyclopedia of Genes and Genomes), COG (Clusters of Orthologous Groups), and Rfam databases (Lu et al., 2019) Using PICRUSt, the 16S rRNA gene sequencing data was matched against a database encompassing established metabolic functions, with the goal of projecting bacterial metabolic capabilities. Given the divergence in the quantity of 16S rRNA gene copies among distinct species, this aspect was considered during the analytical process. To bolster the precision and dependability of our projections, we implemented an adjustment to the original species’ abundance data (Wang et al., 2018).

## Results

### Characteristics of the participants

**Figure 1:**
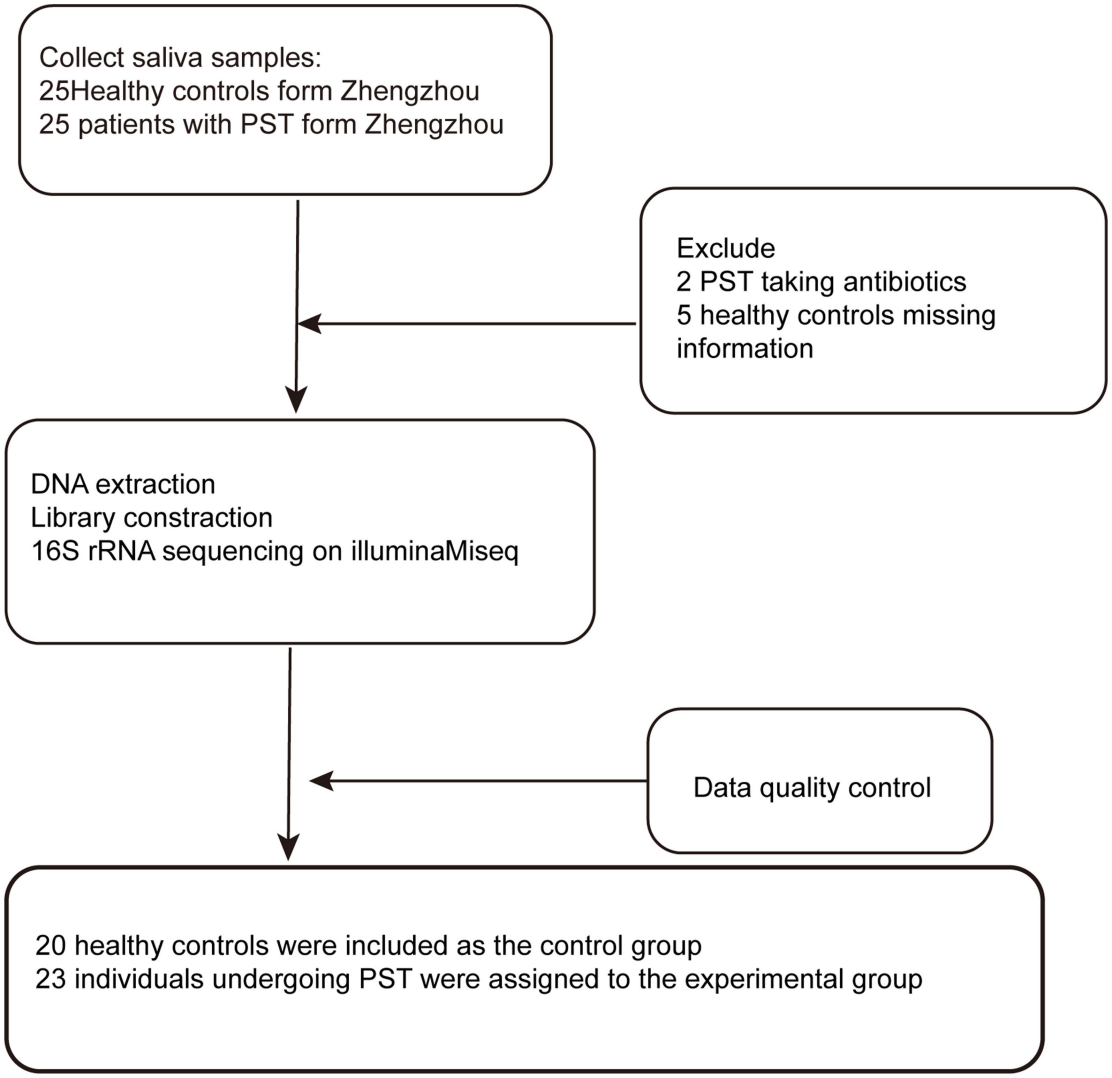
Study design and flow diagram. A total of 50 saliva samples from Zhengzhou were prospectively collected. After a strict pathological diagnosis and exclusion process, 23 PST patients and 20 HCs were included, 20 healthy controls were included as the control group, 23 individuals undergoing PST were assigned to the experimental group. PST, pediatric solid tumors; HCs, healthy controls.

In our research, a sum of 50 saliva samples was procured from individuals residing in Central China. The study included 25 newly diagnosed patients with Pediatric solid tumor and 25 healthy controls who were matched for BMI, gender, and age. After undergoing a rigorous selection and exclusion process (Figure 1). The saliva microbiome of 23 PST patients and 20 HCs as characterized, and microbiome markers were identified.

### The decreased oral microbial diversity in PST

**Figure 2:**
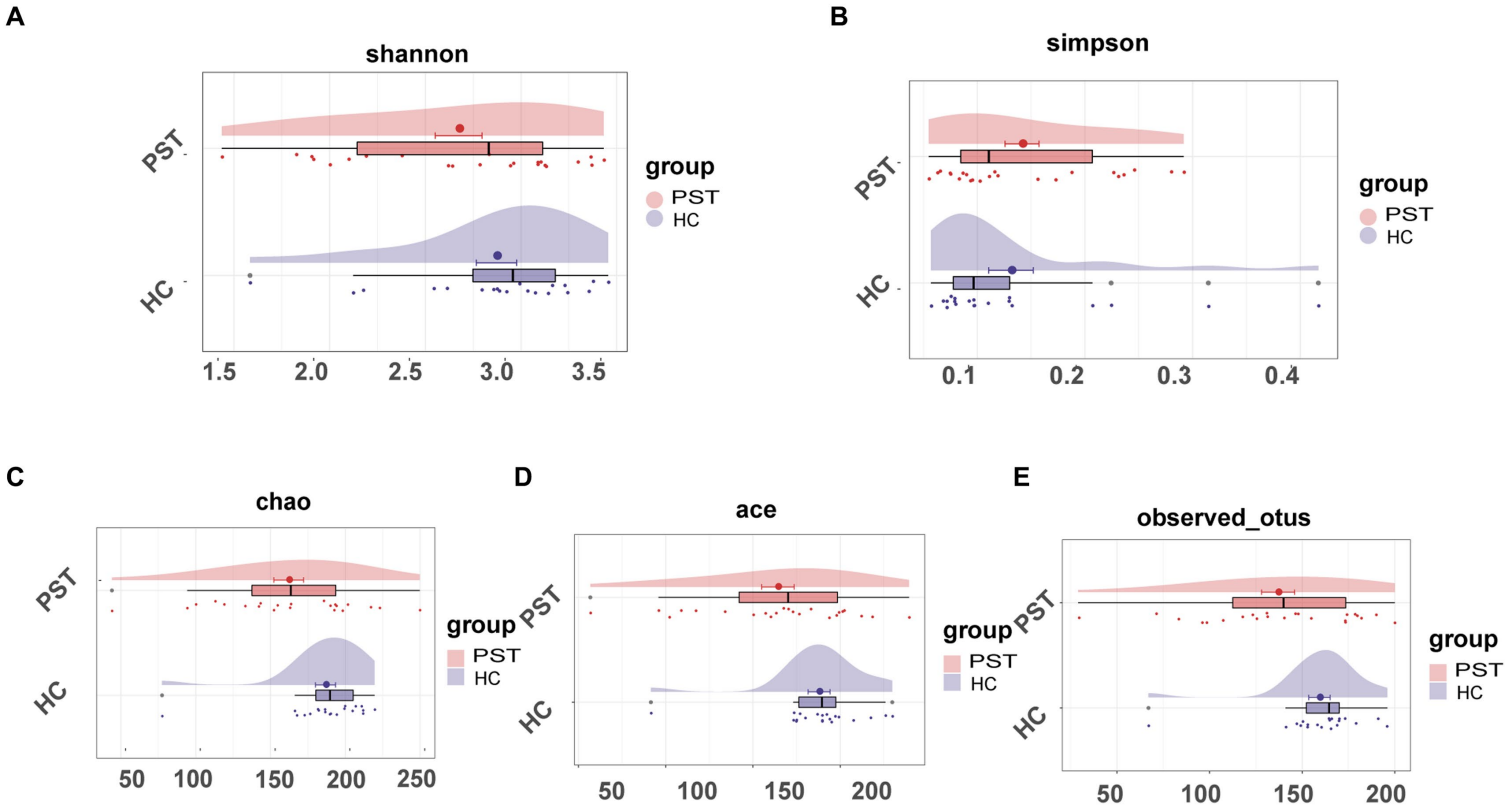
The microbial diversity of patients with PST (*N* = 23) was found to be lower than HCs (*N* = 20). The Shannon **(A)** and Simpson **(B)** indices, the oral microbial diversity of PST patients (red) showed a decreasing trend compared to HCs (blue). The Chao **(C)** and Ace **(D)** indices also indicated that the microbial community richness of PST patients (red) was lower than HCs (blue). This finding was confirmed by the observed OTU **(E)**. PST, pediatric solid tumors; HCs, healthy controls.

In comparison to HCs, oral microbial diversity in PST patients exhibited a notable decline, evident through the evaluation of the Shannon index (Figure 2A) and Simpson index (Figure 2B). Additionally, the Chao index (Figure 2C) indicated that microbial community richness was lower in PST patients than in HCs (*p* < 0.05, Mann–Whitney U test). The validity of this discovery was corroborated by the observed and Ace indices (Figure 2D) as well as the OTU point plot (Figure 2E).

### Disparities in the oral microbiome between PST patients and HCs

**Figure 3:**
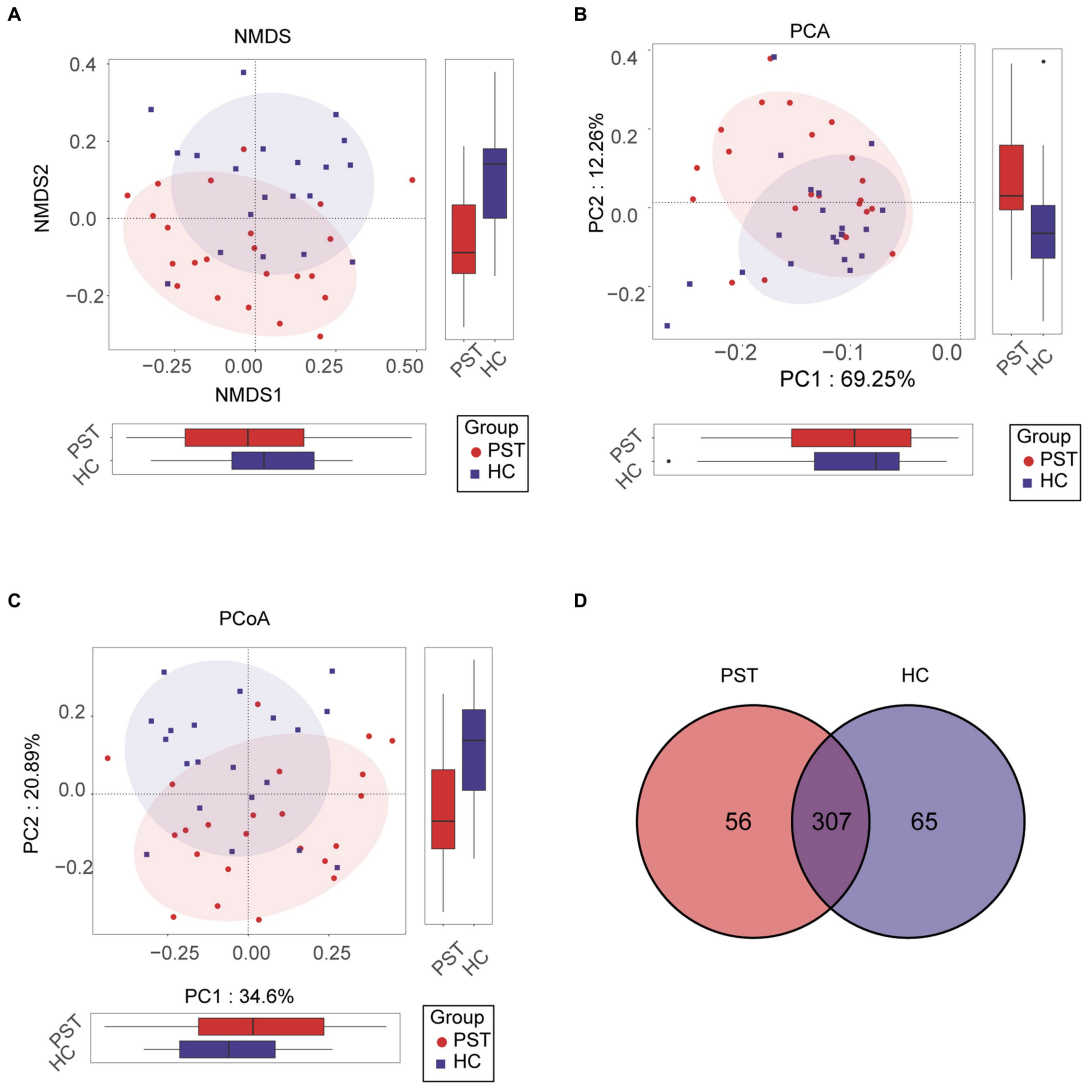
Comparison of beta diversity between PST patients (*N* = 23) and HCs (*N* = 20). The results showed significant differences in the overall oral microbiota composition between PST and HC groups, as indicated by the NMDS analysis **(A)**, PCA plots **(B)**, and PCoA **(C)**. These findings suggested that the microbial community structure of PST patients was different from HCs. The Venn diagram **(D)** demonstrated that out of 428 OTUs, 307 were shared between the two groups. PST, pediatric solid tumors; HCs, healthy controls; OTU, operational taxonomic unit; NMDS, non-metric multidimensional scaling; PCA, principal component analysis; PCoA, principal coordinate analysis.

In order to visualize the microbial community space between samples, a visualization analysis was conducted. To evaluate beta diversity, we employed principal component analysis (PCA), principal coordinate analysis (PCoA), and non-metric multidimensional scaling (NMDS) to investigate the divergence among microbial communities. Results from the unweighted UniFrac NMDS analysis (Figure 3A), unweighted UniFrac PCA with PC1-2 (Figure 3B), and unweighted UniFrac PCoA with PC1-3 (Figure 3C) distinctly exhibited the segregation of samples from PST patients and healthy controls along the NMDS1, PC1, and PC1 axis, underlining an apparent divergence in the overall oral microbiota makeup among the two groups. The primary outcome of the data comparison was the identification of substantial disparities in oral microbial communities between pediatric solid tumor patients and their healthy control counterparts. In addition, a Venn diagram revealed that out of the 428 OTUs, 307 were shared between the PST group and the HC group (Figure 3D). It is worth noting that there were 56 OTUs that were unique to PST out of the 428 OTUs.

### Clustering of operational taxonomic units and taxonomic analysis

**Figure 4:**
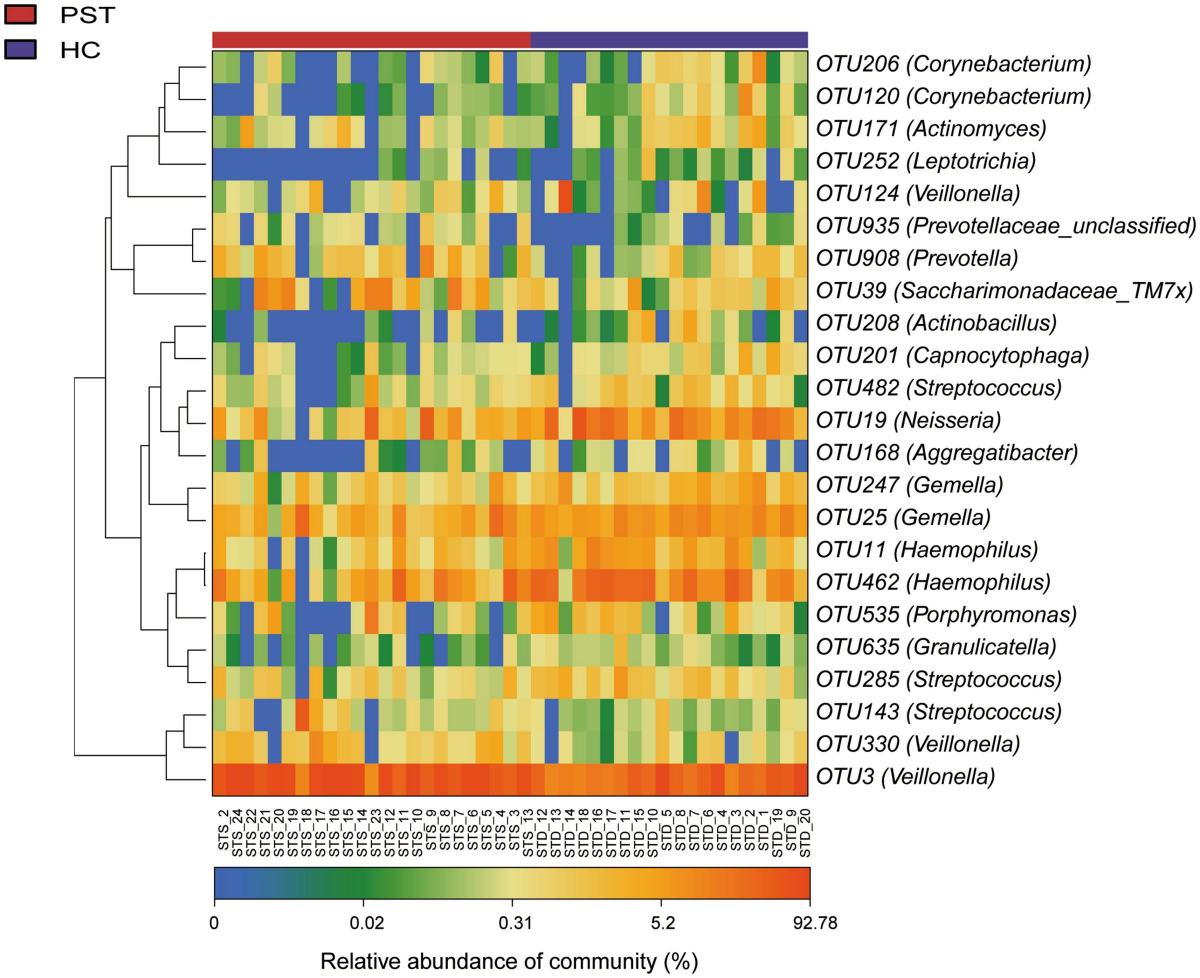
Heatmap displayed the relative abundance differences of OTUs between PST (*N* = 23) and HCs (*N* = 20). The relative abundance of each sample was shown, with red indicating high abundance and blue indicating low abundance. Each row represents an OTU. OTU, operational taxonomic unit; PST, pediatric solid tumors; HCs, healthy controls.

The heat map displaying the differential relative abundance of OTUs between pediatric solid tumor patients and their healthy control counterparts (Figure 4), OTUs with lower relative abundances are represented with shades of blue, while those with higher relative abundances are represented with shades of red.

There is a decrease in a total of 20 species of bacteria in PST patients, including OTU462 (Haemophilus) OTU19 (Neisseria) OTU39 (Saccharimonadaceae_TM7x) OTU908 (Prevotella). However, compared to HCs, the oral microbiota of PST patients shows a significant increase in OTU3 (Veillonella).

### Comparison and composition of the oral microbiome in HCs and PST patients

**Figure 5:**
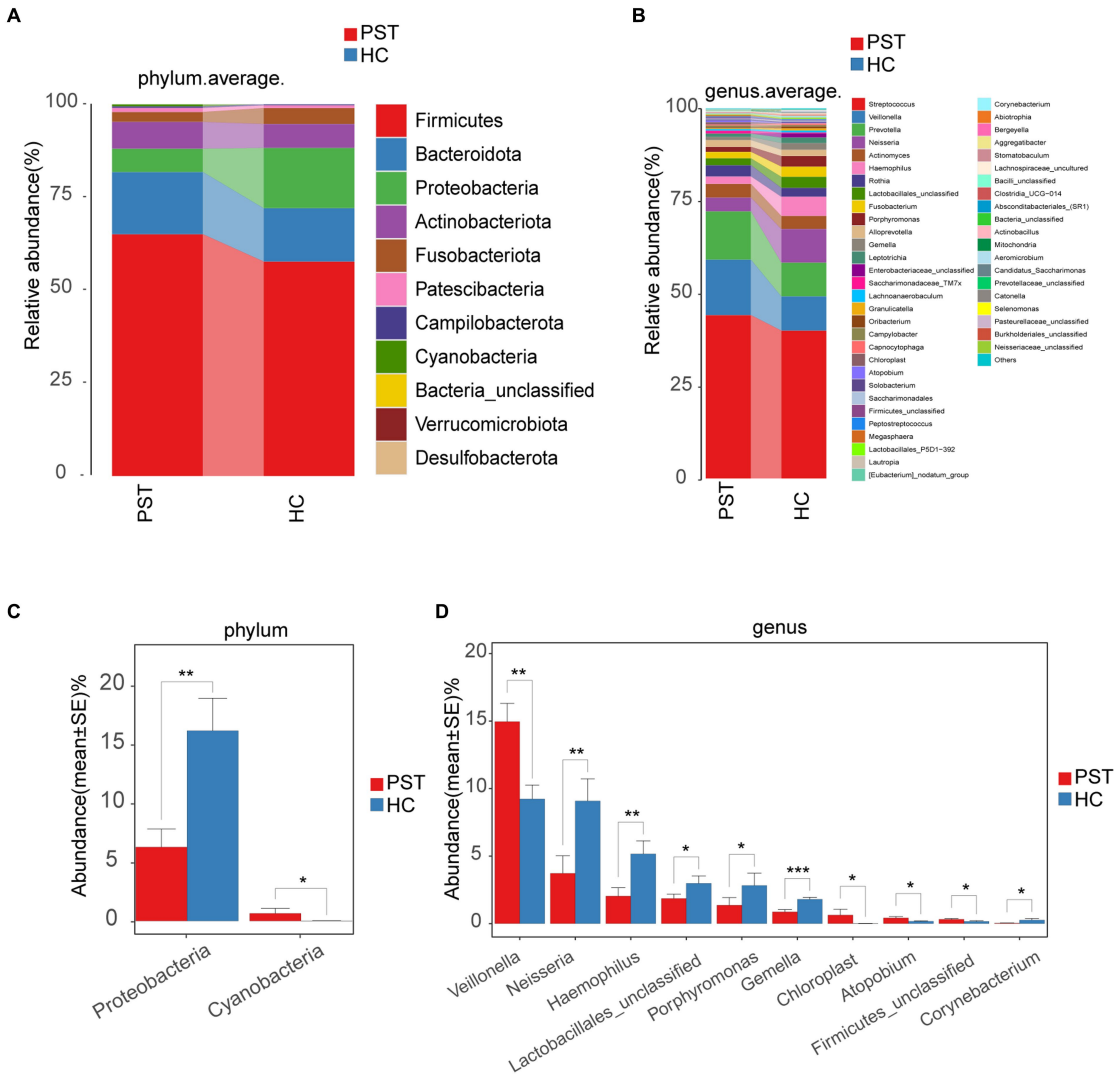
Composition and comparison of the oral microbiome in PST patients (*N* = 23) and HCs (*N* = 20). The composition of the two groups was represented by phylum-level **(A)** and genus-level **(B)** compositions. The relative abundance differences of key bacteria at the phylum and genus levels were shown in figure **(C,D)**. The relative abundance of each bacteria was represented by the mean ± SE. Significance of the differences in relative abundance was evaluated using Wilcoxon rank-sum tests (*p < 0.05; **p < 0.01; and ***p < 0.001). PST, pediatric solid tumors; HCs, healthy controls.

Regarding the comparison and composition of the oral microbiota between PST patients and HCs, the proportional representation of each sample was calculated and graphically presented across various taxonomic levels through OTU annotation. At the phylum level, both groups showed a high average proportion (up to 98%) of Firmicutes, Bacteroidota, Proteobacteria, Actinobacteriota, and Fusobacteriota (Figure 5A). Remarkably, noteworthy dissimilarities within these five principal phyla were apparent when comparing the two groups. Similarly, examining the data at the level of genera, including Streptococcus, Veillonella, Prevotella, Actinomyces, Haemophilus, Neisseria, Rothia, Lactobacillales_unclassified, Fusobacterium, Porphyromonas, Alloprevotella, Gemella, Leptotrichia, and Enterobacteriaceae_unclassified. In both groups, an average of 93% or more of the microbiota was comprised of 15 genera (Figure 5B). In terms of phylum and genus classifications, We observed differences in microbial makeup among the two groups.

Subsequently, a comparison was made between the oral microbiota of PST (*N* = 23) and HC (N = 20) at every level of taxonomy. We employed Wilcoxon rank-sum tests to scrutinize substantial distinctions in microbial makeup between PST patients and healthy controls. In terms of phylum-level analysis, the presence of Proteobacteria was notably diminished in PST patients compared to healthy controls (*p* < 0.01). Conversely, Cyanobacteria showed a significant increase in PST patients (*p* < 0.05) (Figure 5C).

In terms of genus-level analysis, the abundance of Neisseria, Haemophilus, Lactobacillales unclassified, Porphyromonas, Gemella, and Corynebacterium was showed a marked decrease in PST cases in comparison to HCs (*p* < 0.05), while Veillonella, Chloroplast, Atopobium, and Firmicutes unclassified exhibited a significant increase in abundance in PST cases (p < 0.05) (Figure 5D).

In terms of genus-level and phylum-level analysis, the comparisons indicated significant differences, particularly Proteobacteria, Veillonella, Neisseria, Haemophilus and Gemella (*p* < 0.01). Compared with the HCs, Veillonella was significantly increased in the PST group, while Proteobacteria, Neisseria, Haemophilus, and Gemella were all significantly decreased.

### Phylogenetic features of oral microbial communities in PST

Based on Lefse analysis and OTU-based LDA scores, 18 genera have been identified as differentially abundant species in patients with Pediatric solid tumor (PST). Additionally, 29 genera have been identified as dominant in the healthy control group (HC), showing notable distinctions between the two groups.

**Figure 6:**
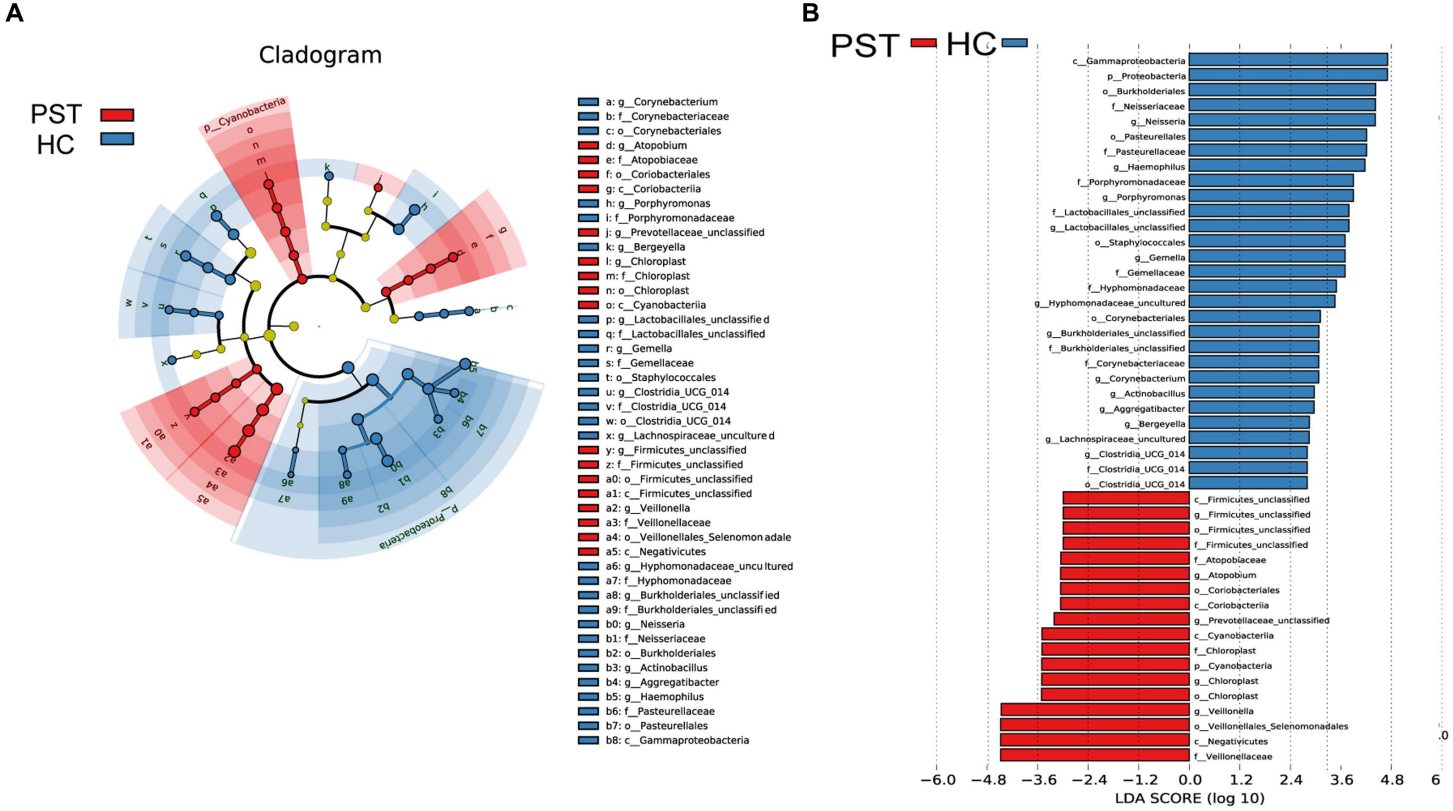
LEfSe and LDA analysis based on OTUs characterize the microbiome between PST and HCs. The phylogenetic tree **(A)** of the oral microbiome associated with PST and HCs was displayed using the LEfSe method. The circles radiating from the center represented the classification levels from phylum to genus. Each level circle represents the classification at that level, and the diameter of the circle represents its relative abundance. Uniformly colored circles indicate no significant difference, while biomarkers with significant differences follow the grouping colors. Species with LDA scores greater than 2 and *p* values less than 0.05 are considered differential species. The histogram of LDA scores **(B)** showed significant differences between the oral microbiome of the two groups. The higher the LDA score, the greater the importance of the microbial biomarker. A default LDA score greater than 2 and p value less than 0.05 is considered a differential species. LDA, Linear discriminant analysis; LEfSe, linear discriminant analysis effect size; OTU, operational taxonomic unit; PST, pediatric solid tumors; HCs, healthy controls.

Graphical depiction of the phylogenetic distribution of oral microbiota linked to PST and HC can be observed in Figure 6A. Figure 6 illustrates histograms of LDA scores computed for the chosen taxonomic clusters, displaying bacteria with significant differences between PST and HC.

Correspondingly, 18 biomarkers, including Veillonellaceae, Negativicutes, Veillonellales Selenomonadales, Veillonella, Chloroplast, Chloroplast, Cyanobacteria, Chloroplast, and CyanobacteriiaStr, were noteworthy enrichment observed in PST patients (*p* < 0.05; LDA score > 2), confirming their status as prevailing genera. Additionally, the prevalence of 29 genera exhibited a notable decrease when compared to the HCs (LDA score > 2; *p* < 0.05).

### Functional analysis of genes

**Figure 7:**
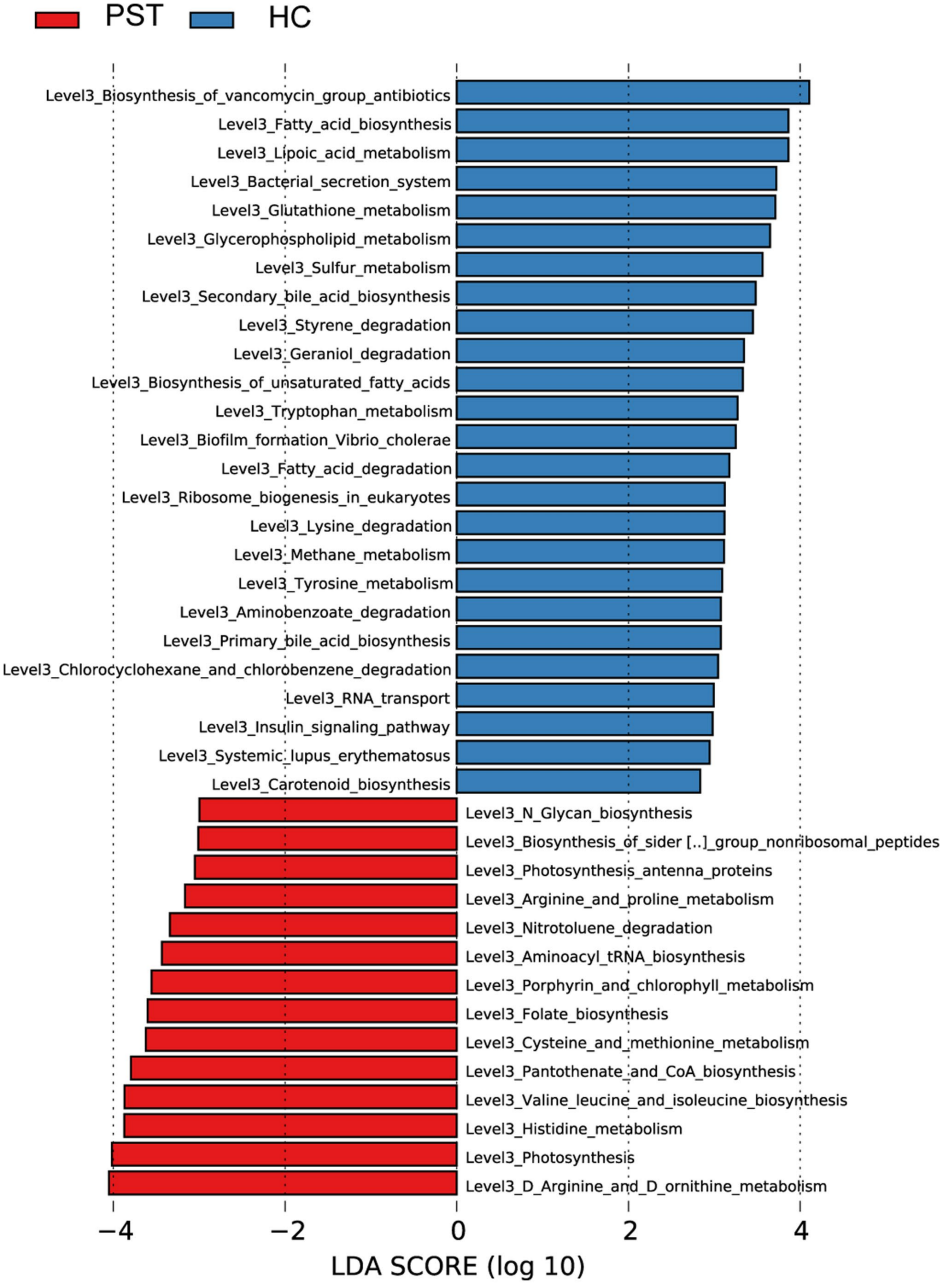
Functional analysis of predicted metagenomes. Displays the predicted functional analysis of the metagenome using the LEfSe method to identify KEGG pathways that were significantly enriched between PST and HCs. The significance of the microbial biomarker was reflected by the LDA score, with a higher score indicating greater importance. A KEGG pathway is considered significantly enriched if it has a default LDA score greater than 2 and a p value less than 0.05. LDA, Linear discriminant analysis; LEfSe, Linear discriminant analysis effect size; KEGG, Kyoto Encyclopedia of Genes and Genomes; PST, pediatric solid tumors; HCs, healthy control.

Graphical depiction of the gene functions’ phylogenetic distribution linked to PST and HC can be observed in Figure 7. Using Lefse LDA scores based on annotation with KEGG orthologous gene clusters (KO), it was found that PST group had 16 gene functions that were significantly dominant, including Aminoacyl tRNA biosynthesis, Amino acid metabolism, Photosynthesis antenna proteins, Arginine and proline metabolism, Biosynthesis of siderophore group nonribosomal peptides, Photosynthesis antenna proteins, Histidine metabolism, Amino acid metabolism, and Cysteine and methionine metabolism.

On the contrary, 29 gene functions were decreased with PST, including RNA transport, Geraniol degradation, Biofilm formation *Vibrio cholerae*, Fatty acid degradation, Styrene degradation, Biosynthesis of vancomycin group antibiotics, and Tryptophan metabolism.

## Discussion

This study presents, for the initial time, the alterations in oral microbiota attributes of patients with Pediatric solid tumor (PST), encompassing aspects of species diversity, composition of communities, and functional gene profiles. The research particularly zeroes in on the attributes of oral microbiota among PST patients from the central region of China. Sequencing of oral microbiota was performed using the 16S rRNA gene on samples obtained from 23 PST patients and 20 paired healthy controls (HCs) from the central region of China. Surprisingly, There was a notable decrease in oral microbiota diversity among PST patients as compared to HC. Interestingly, similar alterations also occur in adult disease, for example, patients diagnosed with liver cancer and cirrhosis also exhibit a decline in microbial diversity (Effenberger et al., 2023). The precise factors accountable for the divergence in diversity among gut and oral microbial communities remain unknown (Rao et al., 2021). The occurrence of diseases may be associated with an increase or decrease in microbial diversity, which may vary depending on the specific disease. In patients with autoimmune hepatitis (AIH), a marked rise in Veillonella was observed alongside a simultaneous reduction in Streptococcus within the oral microbiota, as contrasted with the healthy controls (HCs) (Abe et al., 2018). In the high Acute Pancreatitis (AAC) score group, a declining pattern in microbial diversity was observed, characterized by notably reduced species abundance in comparison to the low AAC score group (Bao et al., 2023). The chronic pancreatitis group exhibited the lower microbial diversity than healthy controls (Chen et al., 2023). Patients with breast cancer demonstrated lower intestinal microbial diversity compared to the healthy controls (Thu et al., 2023). Through PCoA, PCA and NMDS analyses, evident disparities in the composition of the oral microbiota community were evident when comparing PST patients and HC. The application of LefSe analysis and the subsequent LDA scores unveiled marked distinctions in the composition of oral microbiota between two groups. A total of 18 genera exhibited differential abundance in PST patients, conversely, in the HC group, 29 genera were identified as prevailing, signifying notable dissimilarities between the two groups.

With the advancement of metagenomics research, our understanding of the oral microbiota has deepened, and their relationship with diseases has become increasingly clear. For instance, dysbiosis of the oral microbiota can lead to various systemic diseases, such as inflammatory bowel disease (Read et al., 2021). The oral microbiota exerts its influence on oral squamous cell carcinoma (OSCC) through direct metabolism of carcinogens and inflammatory effects (Irfan et al., 2020). Oral bacteria can convert alcohol into acetaldehyde, which is a carcinogenic agent that promotes the malignant transformation of the mucous membranes in the head and neck region (Galvão-Moreira and da Cruz, 2016). Carrying oral pathogens, such as *Porphyromonas gingivalis* and *Aggregatibacter actinomycetemcomitans*, is significantly associated with a higher risk of pancreatic cancer. Conversely, the presence of bacteria from the Firmicutes phylum, particularly the genus Lactobacillus, is associated with a reduced risk of pancreatic cancer (Fan et al., 2018). Compared to the healthy control group, levels of Capnocytophaga and Veillonella in the saliva of lung cancer patients, including squamous cell carcinoma (SCC) and adenocarcinoma (AC), are significantly elevated (Yan et al., 2015). *Porphyromonas gingivalis* is associated with an increased risk of Alzheimer’s Disease (Elwishahy et al., 2021).

Furthermore, the oral microbiota holds the potential to serve as a diagnostic tool in clinical settings. As research progresses, the association between oral microbiota and diseases will also gain greater clarity. Utilizing oral microbiota as emerging biomarkers for colorectal cancer screening (Rezasoltani et al., 2022) The aforementioned research discusses the connection between oral microbiota and adult diseases. In the case of children, salivary proteins and microbial communities serve as biomarkers for early assessment of dental caries risk (Hemadi et al., 2017). The oral microbiome is closely associated with children’s health (Xiao et al., 2020). Oral microbiota are linked to obesity in children and adolescents (Mameli et al., 2019). However, the oral microbiota changes in pediatric solid tumor patients have not been studied yet. This is precisely the purpose of our research.

We speculate that oral microbiota detection could emerge as a novel and efficient tool for distinguishing between HCs and PST patients. It offers advantages such as low invasiveness, cost-effectiveness, high acceptability, and diagnostic efficacy. Therefore, we believe that this research is highly valuable and significant.

## Conclusion

This study presents the initial assessment of oral microbiota among Pediatric solid tumor patients (PST). Importantly, targeted biomarkers of the oral microbiota have the potential to act as potent and non-intrusive diagnostic instruments for Pediatric solid tumors (PST).

## Data availability statement

The datasets presented in this study can be found in online repositories. The names of the repository/repositories and accession number(s) can be found below: NCBI, PRJNA991748.

## Ethics statement

The studies involving humans were approved by all participants signed written informed consent. The ethics certificate shall be reviewed and approved by the Ethics Committee of the First Affiliated Hospital of Zhengzhou University. The studies were conducted in accordance with the local legislation and institutional requirements. Written informed consent for participation in this study was provided by the participants’ legal guardians/next of kin.

## Author contributions

XiC: Conceptualization, Data curation, Formal analysis, Funding acquisition, Writing – original draft, Writing – review & editing. XD: Investigation, Methodology, Project administration, Resources, Writing – review & editing. XuC: Software, Supervision, Validation, Visualization, Writing – review & editing. RF: Software, Supervision, Validation, Visualization, Writing – review & editing. JP: Software, Supervision, Validation, Visualization, Writing – review & editing. ZW: Conceptualization, Data curation, Formal analysis, Funding acquisition, Writing – original draft, Writing – review & editing.
