# Supplementary material for: Oral microbiome characteristics in patients with pediatric solid tumor
Source: Front Microbiol. 2024 Jan 5;14:1286522. doi: 10.3389/fmicb.2023.1286522 (PMC10797044; doi:10.3389/fmicb.2023.1286522)
Supplement: Supplementary file 1 [file Table_1.DOC]

The data has been successfully uploaded and reviewed. The SRA accession number has been generated: PRJNA991748, with a release date of June 1, 2027, and it can be modified at any time. Below is the private link for the project, accessible for reviewers during the confidential pre-publication stage. Feel free to share it with the journal editor for review if necessary.

<https://dataview.ncbi.nlm.nih.gov/object/PRJNA991748?reviewer=2ibrm0rr0vc3khcbmrohjqf3t4>
